# Life Cycle Assessment of Chemical Upcycling of Postconsumer Polyethylene Terephthalate to Kevlar Polymer

**DOI:** 10.1021/acssuschemeng.5c04073

**Published:** 2025-10-29

**Authors:** Jaewon Han, Preeti Nain, Richard-Joseph L. Peterson, Elanna P. Neppel, Annick Anctil

**Affiliations:** † Department of Civil and Environmental Engineering, 3078 Michigan State University, East Lansing, Michigan 48824, United States; ‡ Department of Chemical Engineering, 3078 Michigan State University, East Lansing, Michigan 48824, United States

**Keywords:** plastic recycling, chemical upcycling, polyethylene terephthalate, kevlar, life cycle assessment, green chemistry

## Abstract

Due to its low cost and chemical stability, poly­(ethylene terephthalate) (PET) is widely used in single-use items, such as plastic bottles and packaging materials. The high demand has resulted in a significant amount of discarded PET waste, underscoring the need for effective recycling strategies. One promising approach is PET chemical upcycling, but it requires evaluation of the environmental impact of the entire process, from waste collection to polymer synthesis. This study, for the first time, uses life cycle assessment (LCA) to assess the environmental impact of chemical upcycling of postconsumer PET to Kevlar polymer. The study models the life cycle inventory of chemical upcycling via PET ammonolysis and hydrolysis, developing scenarios based on the environmental hotspots of each chemical process for future optimization. The results show that upcycled Kevlar (15.22 kg CO_2_ eq/kg Kevlar) has lower impacts than virgin Kevlar polymer production, with a 15% reduction in global warming potential (GWP), 30% in cumulative energy demand (CED), 1% in ecotoxicity, and 40% in fossil fuel depletion. Hotspot analysis identifies monomer preparation as the most impactful process, particularly due to the use of solvents such as chloroform and diethyl ether. Chloroform, used in the p-phenylenediamine (PPD) preparation process, is one of the largest contributors, accounting for approximately 20.8% of GWP and 12.1% of ecotoxicity. The best-case scenario, which replaces potassium hydroxide with sodium hydroxide and reduces solvent consumption for purifying and extracting monomers, results in a 19.9% decrease in GWP and a 21.1% reduction in costs compared to those of the baseline. These results highlight that upcycled Kevlar has the potential to reduce the environmental burden of Kevlar production by utilizing PET waste. However, achieving adoption on an industrial scale requires targeted process optimization, addressing process hotspots, and replacing conventional solvents with greener alternatives.

## Graphical abstract

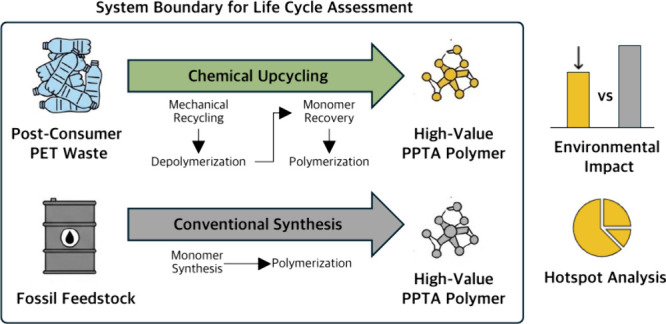

## 1 Introduction

Global plastics production reached 359 million tons in 2018 and is projected to increase further, reaching 1.1 billion tons by 2050.^1,2^ One of the most commonly used plastics is polyethylene terephthalate (PET) due to its lightweight nature, high chemical stability, and outstanding barrier properties.^3^ Owing to these features, PET is used for bottled beverages, food packaging, textiles, medical devices, and electrical applications.^4^ The widespread use of plastics across various industries, their short lifespan as single-use items, and inadequate disposal practices have led to a substantial accumulation of discarded PET plastic in the solid waste stream.^5^ The growing plastic waste has emerged as one of the most pressing environmental challenges. Only 10% of the total plastic waste collected is recycled, while most is dumped in landfills or left to accumulate in natural environments.^6^

Recycling plastic waste offers multiple benefits, minimizing waste, conserving petroleum resources, and ensuring that plastics are continuously reintroduced into the production process in a closed loop.^4,7^ Currently, mechanical recycling of PET waste is the most widely adopted approach, though it often results in the deterioration of material performance.^8–10^ Compared with mechanical recycling, chemical recycling of PET breaks down the polymer into monomers, which can be used again for polymerization.^11^ Common chemical recycling technologies for waste PET include depolymerization through methanolysis, glycolysis, ammonolysis, and hydrolysis.^12–14^ With respect to chemical recycling, previous studies are limited to monomers such as bis­(hydroxyethyl) terephthalate (BHET) and terephthalic acid, utilizing various solvents, including water, alcohol, and amines, to dissolve PET.^4^ Despite variations in products, the GWP values for depolymerization consistently fall within a certain range, with BHET having a GWP of 2.59 kg CO_2_ eq/kg^15^ and DMT ranging from 2.71 to 4.18 kg CO_2_ eq/kg.^16^

Within the realm of chemical recycling technologies, depolymerization via ammonolysis and hydrolysis dissolves PET into monomers, which can be subsequently upcycled into a value-added polymer such as Kevlar.^17^ Kevlar, developed by DuPont in 1965, was the first para-aramid fiber, and this high-performance fiber was first commercially used in the automobile industry.^18^ The Kevlar fiber and its composites possess a very high ratio of tensile to compression strength, finding application in military and aerospace applications.^19^ Globally, the annual production capacity for recycled PET (rPET) is estimated at approximately 20 million tons, notably exceeding the global production capacity for para-aramid fibers (Kevlar) of approximately 160,000 tons per year.^20^ Approximately 1.62% of recycled PET production capacity would be required to meet global Kevlar demand. This demonstrates the feasibility of utilizing this abundant waste stream as a feedstock for high-value applications. However, as chemical upcycling is a new technology, it is important to assess it for its environmental impact.^10^

Life cycle assessment (LCA) is commonly used to evaluate the potential environmental impacts of a product or new technology based on ISO 14040:2006 and ISO 14044:2006.^21,22^ Applying LCA modeling to plastic recycling provides a comprehensive understanding of the environmental impacts and allows for decision-making on improving the recycling process.^10,23^ Most previous LCA studies considered mechanical recycling of PET, showing its low environmental impacts, mainly due to the avoidance of virgin PET resin production and reduced fossil resource consumption.^24–26^ Previous LCA studies on chemical recycling show a wide range of environmental impacts, depending on the system boundaries and technologies considered.^27^ However, LCA studies of PET chemical recycling are limited to depolymerization into monomers, while a significant gap remains in assessing the environmental impact of upcycling PET-derived monomers into higher-value polymers, such as Kevlar.^17,18^ Only one study evaluated the life cycle impacts of Kevlar, but inventory and modeling details were not provided.^28^ To commercialize PET waste into Kevlar, understanding the environmental impacts at various process stages and solvent consumption is required.

To fill the above research gaps, this study aims to (i) model the life cycle inventory of waste PET upcycling to the Kevlar polymer process via ammonolysis and hydrolysis and virgin Kevlar polymer production, (ii) assess the environmental impacts associated with the chemical upcycling process and compare them with the virgin polymer production, and (iii) propose scenarios to reduce the environmental impact of the upcycling process by replacing or reducing chemical consumption.

## 2 Methodology

### 2.1 LCA Goal and Scope

The environmental impact of chemical processes of upcycled Kevlar from PET and fossil-based virgin polymer production is quantified by LCA based on the ISO 14040 and 14044 standards.^22^ The chemical upcycling process involves depolymerizing PET flakes through ammonolysis and hydrolysis to obtain Kevlar polymer precursors. The study also aims to develop a life cycle inventory for the chemical upcycling process and identify environmental hotspots in each step for future optimization. The comparison assumes identical purity and quality between upcycled Kevlar and the virgin Kevlar polymer. As the chemical upcycling is currently at the laboratory scale, the life cycle inventory is modeled by scaling up data from lab experiments using available data from patents and published studies.

The functional unit for this study is 1 kg of Kevlar production. The system boundary is shown in Figure 1, illustrating the chemical upcycling process, starting from mixed plastic waste collection to product polymer synthesis.

**Figure 1:**
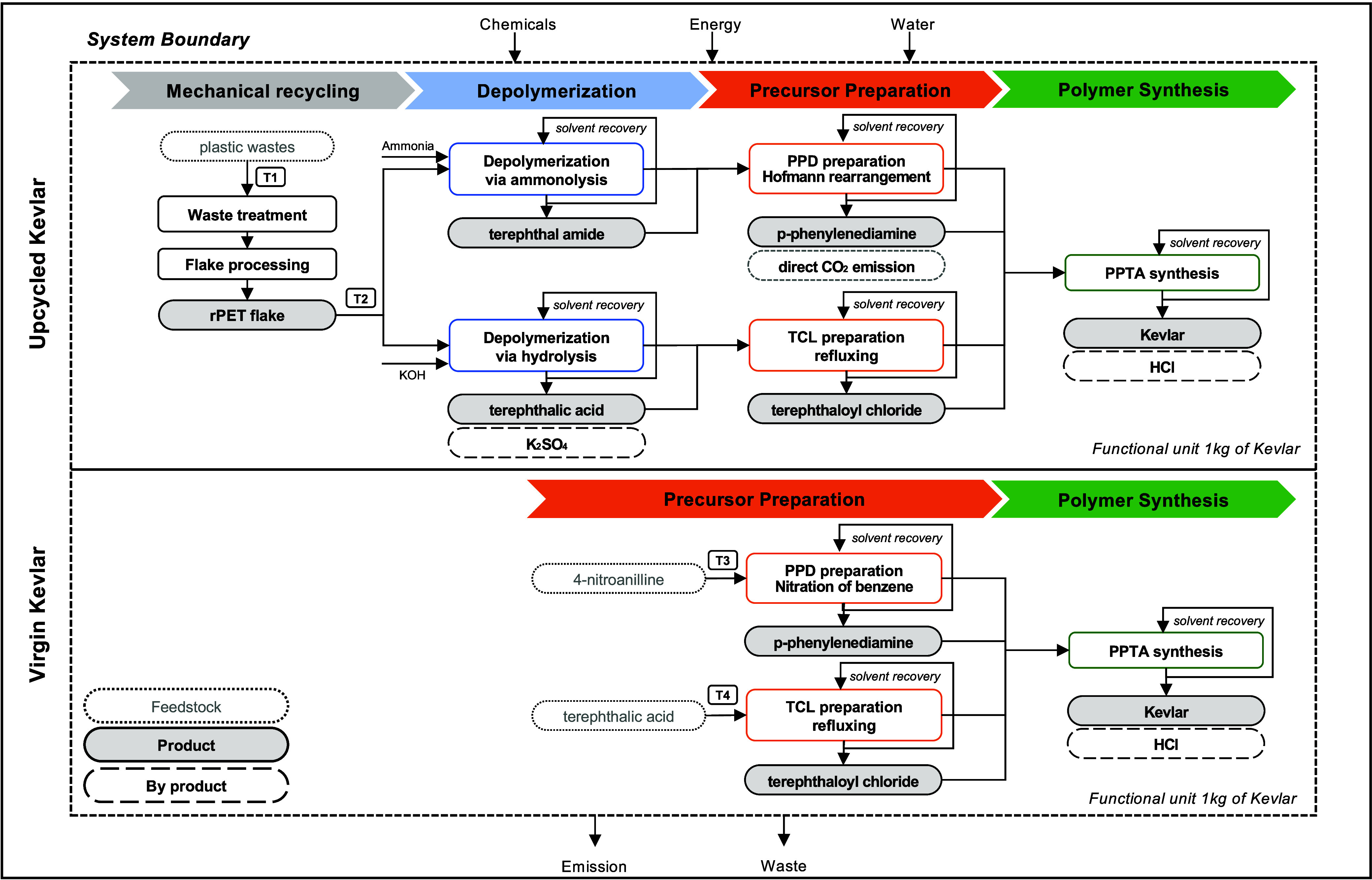
System boundary of chemical upcycling of postconsumer PET to Kevlar polymer and virgin Kevlar production. T1-T4 correspond to the transportation of intermediate materials to the processes.

### 2.2 Allocation for Open-Loop Recycling

An open-loop allocation procedure applies to product systems where the material is recycled into other product systems and the material undergoes a change to its inherent properties.^22^ The virgin material production, recycling, and final waste management activities involved in open-loop recycling are multifunctional processes since they provide different product life cycles with different functions.^15^ Although various approaches have been proposed, there has been no standardized allocation methodology for open-loop recycling.^26,29^

#### 2.2.1 Cut-Off Approach

The cutoff approach is most commonly applied to avoid double-counting burdens between product systems and to avoid assumptions on market conditions.^29,30^ In the cutoff approach, the environmental burdens from material production and waste generation are fully assigned to the original product, and recycling starts from waste treatment.^30^

#### 2.2.2 50/50 Approach

The 50/50 approach allocates 50% of the burdens of virgin material production and the recycling process to the virgin product and the recycled product's life.^31^ The rationale underlying this method is that both supply and demand for recycled materials are necessary to enable recycling.^32^

The equations for the environmental impact (EI) are

$$\mathrm{First}\;\mathrm{life}(\mathrm{recycling})={\mathrm{EI}}_{\mathrm{virgin}\;\mathrm{production}}+0.5\times {\mathrm{EI}}_{\mathrm{recycling}\;\mathrm{process}}-0.5\times {\mathrm{EI}}_{\mathrm{virgin}\;\mathrm{production}}$$

$$\mathrm{Second}\;\mathrm{life}(\mathrm{use}\;\mathrm{of}\;\mathrm{recycled}\;\mathrm{material})=0.5\times {\mathrm{EI}}_{\mathrm{virgin}\;\mathrm{production}}+0.5\times {\mathrm{EI}}_{\mathrm{recycling}\;\mathrm{process}}+{\mathrm{EI}}_{\mathrm{second}\;\mathrm{life}\;\mathrm{production}}$$

#### 2.2.3 Waste Valuation

The waste valuation approach considers waste as a valuable resource. The environmental impact of virgin PET production should be allocated to the second life based on the economic allocation factor, since mass allocation cannot be assumed due to different products from each life cycle. The allocation factor (AF) is defined as the ratio of the market value of baled PET waste to the market value of virgin PET bottle grade resin.

$$\mathrm{Second}\;\mathrm{life}(\mathrm{use}\mathrm{of}\;\mathrm{recycled}\;\mathrm{material})={\mathrm{EI}}_{\mathrm{cut}-\mathrm{off}}+\mathrm{AF}\times {\mathrm{EI}}_{\mathrm{virgin}\;\mathrm{production}}$$

The prices of baled PET waste and virgin PET bottle grade resin are obtained from the monthly prices of North America plastics published by A Resource Recycling Inc. and Business Analytiq for the last five years.^33,34^ In general, the AF is in the range of 11–51%, and the average AF, approximately 24.5%, is assumed to shift the environmental burden of virgin PET to the upcycling process.

In this study, we started the analysis with the cutoff approach, where the system boundary of PET chemical upcycling begins with the collection of mixed waste to produce Kevlar. In addition, two alternative approaches, 50/50 and the waste valuation approach, are introduced to compare the allocation methodology for open-loop recycling.

### 2.3 Upcycled Kevlar

#### 2.3.1 Mechanical Recycling

Mechanical recycling of mixed plastic waste produces clean recycled PET flakes for upcycling. The plastic waste is collected at material recovery facilities to be pressed, sorted, and cleaned. The PET flakes undergo a pretreatment process to remove contaminants, including labels, adhesives, and residual liquids, ensuring a high-purity feedstock. The flakes are extruded by using a melt filter to remove further impurities. The study does not consider pellet processing since the upcycling process does not require a pellet shape for the reaction. Transportation of plastic waste includes curbside, consumer drop-off, CRV drop-off, and commercial collection.^35^

#### 2.3.2 Depolymerization and Precursor Preparation

Part of chemical recycling includes PET polymer being broken down into monomers via various depolymerization technologies.^15,36^ Two routes for chemically recycling PET are investigated in this study: depolymerization through ammonolysis and hydrolysis to yield terephthalamide and terephthalic acid. The foundational chemical processes for PET depolymerization via ammonolysis and hydrolysis, along with subsequent polymerization steps, are established in prior research.^37,38^ During the ammonolysis reaction, bis­(2-hydroxyethyl) terephthalate reacts with ammonia in the presence of excess ethylene glycol to form terephthalamide at 125 °C. Terephthalic acid is obtained through base-catalyzed hydrolysis of PET at 50 °C with sodium hydroxide and ethanol.^37^ These monomers can be further reacted to prepare two precursors for Kevlar (*p*-phenylene terephthalamide, PPTA) synthesis: *p*-phenylenediamine (PPD) and terephthaloyl chloride (TCL). PPD is prepared through the Hofmann rearrangement of terephthalamide obtained from PET ammonolysis. TCL is prepared by chlorinating the terephthalic acid obtained from PET hydrolysis with excessive thionyl chloride and dimethylformamide at 80 °C from PET hydrolysis.

#### 2.3.3 Kevlar Polymer Synthesis

Virgin Kevlar production uses petrochemical-derived monomers, while upcycling uses waste PET, differing only in feedstocks. To synthesize PPTA, PPD and TCL monomers obtained from PET ammonolysis and hydrolysis are added to N-Methyl-2-Pyrrolidone (NMP) solution with calcium chloride to improve the molecular weight of the polymer.^37,39^ It undergoes a condensation reaction between PPD and TCL, producing hydrochloric acid as a byproduct. Traditionally, these monomers are derived from petroleum. Using monomers derived from waste, as previously described, adds value to the waste and reduces the need for petrochemicals in polymer synthesis.

### 2.4 Life Cycle Inventory

#### 2.4.1 Chemicals Inventory

The chemical consumption data for modeling the upcycling process are mainly derived from original patents, published literature, and the Ecoinvent v3.10 database. Publicly inaccessible chemical inventory data are estimated using stoichiometric calculations. Solvent regeneration with a 95% recovery rate and byproduct recovery rate of 68% is assumed for all chemical processes.^40,41^ The waste from chemical processes is calculated based on the system mass balance and treated through hazardous waste incineration, which is the most common waste management for industrial chemical waste.^42^ The inventory of important parameters and assumptions of the process is summarized in Table 1. Detailed assumptions and life cycle inventory for intermediate products are provided in Tables S1 and S2. Additionally, the mass balance diagram for 1 kg of upcycled Kevlar is available in Figure S1.

**Table 1:** Assumptions on Inventory Data for the Industrial Scale Production of PET Chemical Upcycling to Kevlar Polymer

**Process**	**Inventory**	**Assumption**	**Reference**
Mechanical recycling	yield	85%	^43^
Depolymerization to terephthal amide	yield	87%	^44^
	NH_3_	1:2 wt % to PET flake	^38^
Depolymerzation to terephthalic acid	yield	93%	^45^
	KOH	same mole with 0.5 kg NaOH/kg PET	^46^
	EG	1:1 mass ratio to water	^45^
	H_2_SO_4_	0.36 mL/g	^38^
PPD preparation	yield	90%	^38^
	NaOH	2.22 kg/kg PPD	^47^
	Cl_2_	1.32 kg/kg PPD	^47^
	DI water	3 M NaOH	^38^
	chloroform	2:1 vol %	^38^
TCL preparation	yield	96%	^37^
	TCL	7 mL/3 g acid	^48^
	diethyl ether	2.5:1 vol %	^37^
PPTA synthesis	NMP	16 wt % to CaCl_2_	^37^
	DI water	2:1 vol % to NMP	^37^

#### 2.4.2 Energy Consumption

Since the upcycling process is an emerging technology, the energy consumption is scaled up from the laboratory work done by a coauthor to an industrial scale based on the scaling-up method.^41^ The calculation involves the energy required for heating during reaction, stirring, distillation, drying, and solvent regeneration. The inputs for the equation, such as the mass of the reaction mixture, are scaled up from the laboratory process. The specific heat capacity of solvents for each process is calculated by eqs 1 and 2.

$$Q_{\mathrm{react}}=\frac{Q_{\mathrm{heat}}+Q_{\mathrm{loss}}}{\eta_{\mathrm{heat}}}$$ 1

$$Q_{\mathrm{react}}=\frac{C_{p}\times m_{\mathrm{mix}}\times (T_{r}-T_{0})+A\times \frac{k_{a}}{s}\times (T_{r}-T_{\mathrm{out}})\times t}{\eta_{\mathrm{heat}}}$$ 2

The heating energy required consists of the energy needed to raise the reaction mixture to a specific temperature and maintain it throughout the reaction (*Q*_react_). This is calculated as the sum of the energy for raising the temperature (*Q*_heat_) and heat loss on the reaction surface (*Q*_loss_) divided by the reactor efficiency (η_heat_). Considering the level of the new technology, we calculate the energy consumption for a 100L and 500L batch reactors, and a 10% higher reactor volume compared to the reaction mixture volume is applied based on expert estimates. The heat can be calculated with heat capacity (*C*_p_), the reaction mixture (*m*_mix_), and the gap in reaction temperature (*T*_r_) and starting temperature (*T*_0_) according to eq 2. To calculate the heat loss, the surface area of the reactor (*A*), the thermal conductivity of the insulation material (*k*_a_), the thickness of the insulation (*s*), the temperature difference between the inside and outside of the reactor (*T*_r_ – *T*_out_), and the time of the reaction (*t*) are considered. Detailed information for calculating energy consumption and reaction conditions is available in Tables S3 and S4.

#### 2.4.3 Transportation

The transportation modeling involves collecting mixed plastic waste to the material recovery facility (MRF) and transporting clean PET flakes to the manufacturing plant for upcycling. Waste is collected curbside, through consumer drop-off, deposit, and commercial collection, primarily by truck.^43^ The study assumes mechanical recycling of PET waste is located at PolyQuest, a PET reclaimer in South Carolina,^49^ and all chemical processes are located in Dupont’s Virginia plant.^50^ Those locations were selected because Dupont is the largest Kevlar production plant in the US, and PolyQuest is the nearest major PET reclaimer from the plant to source the recycled PET flake.

The amount of mixed waste and clean flake is calculated based on the yield of each process to produce 1 kg of Kevlar polymer with 91% sorting efficiency and 85% flake processing loss.^43^ Transportation of raw chemicals for virgin Kevlar production is done by truck, and the distance is 224 km for basic chemicals based on the 2017 commodity flow survey.^51^

The life cycle inventory of the chemical upcycling process, including intermediate products and virgin Kevlar production, is provided in Tables S1 and S2. Inventory data for each process refer to the input and output for 1 kg of production of each process.

### 2.5 Life Cycle Impact Assessment

Environmental data of materials, energy, and emissions is obtained from the Ecoinvent 3.10,^52^ following the methods outlined in ISO 14040 and 14044 standards^21,22^ to analyze four impact categories in the North American context: global warming potential (GWP), cumulative energy demand (CED), ecotoxicity, and fossil fuel depletion. TRACI 2.1 v1.09 is used for GWP (kg CO_2_ equiv), ecotoxicity (CTUe), and fossil fuel depletion (MJ surplus), and Cumulative Energy Demand v1.12 is used for CED (MJ). GWP is selected to assess climate change impacts, and CED is used to quantify the total energy demand of the process. Ecotoxicity is included to account for environmental risk from chemical discharges, such as chlorinated chemicals, to the ecosystem.^53,54^ Fossil fuel depletion is considered because both PET plastics and Kevlar are produced from fossil-based petrochemical feedstocks. Ecotoxicity is evaluated by considering the toxicity of chlorinated chemicals to the ecosystem.

### 2.6 Monte Carlo Analysis

Uncertainty arises in an LCA study when the contributions associated with input data are not precisely known and when there is variation within the input data.^55^ Uncertainty analysis is crucial in LCA studies to enhance the robustness of the results. The Monte Carlo approach is the most commonly used approach and is integrated in commercial LCA software, such as SimaPro.^56^ The simulation generates random numbers, which are then used to represent a specific probability distribution.^56^

In our study, the simulation was performed using SimaPro’s Monte Carlo simulation tool, with 1000 iterations at a 95% confidence level to estimate uncertainties.^57,58^ Given the inherent variability and limited data for the emerging upcycling process on a lab scale, these foreground parameters were modeled using a log-normal distribution, and the squared geometric standard deviation is set to 1.2.^57^ Background processes from the ecoinvent database maintained their embedded uncertainty distributions.

### 2.7 Scenario Analysis

In this section, the study examines various scenarios to optimize the chemical upcycling process of postconsumer PET, aiming to minimize environmental impacts. These scenarios encompass the exploration of alternative chemicals, adjusting chemical consumption, and the recovery of valuable byproducts based on the results of hotspot analysis. Table 3 shows the baseline assumptions and modifications for chemicals categorized by specific processes. Leveraging LCA and hotspot analysis, we developed eight scenarios (Table 2) to identify the reduction potential.

**Table 2:**
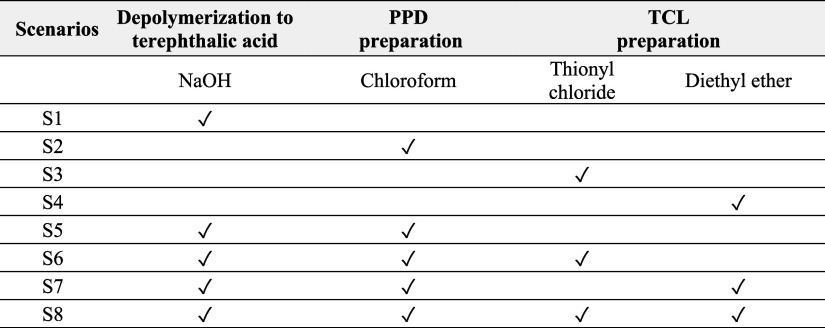
List of Scenarios Evaluated, Including Single Factor Modifications and Combinations

In the process of PET depolymerization to terephthalic acid, NaOH can replace KOH, considering its natural abundance and easier extraction process.^59^ Optimizing solvents is another promising approach to lowering the environmental impacts of PET upcycling. On an industrial scale, solvent volume can be reduced by employing a batch reactor with improved mixing and higher concentration gradients. Therefore, a minimum 1:1 vol % extraction solvent is applied based on the extraction experiment tested in the lab. Another main contributor, thionyl chloride consumption, can be minimized by utilizing a reflux setup, which condenses and recycles the solvent back into the reaction mixture in industrial plants.^60^ The modification on thionyl chloride consumption is made based on the average of the molar ratio applied in the patent and the theoretically required molar ratio. However, the scenario analysis excluded modification on the NMP solution for the polymer synthesis, as the baseline scenario assumed the maximum 16 wt % of ClaCl_2_ to NMP ratio.^61^

### 2.8 Cost Analysis

This section estimates the cost associated with consumed chemicals, solvents, and electricity over the product’s life cycle based on the modeled LCI. Operating, capital expenditures, and labor costs are excluded. The purpose of this cost analysis is to provide the economic implications of the chemical upcycling technology by comparing it with the conventional chemical process, focusing on the operational costs (utility and material consumption). The monomers derived from waste PET via ammonolysis and hydrolysis (PPD and TCL) are chemically identical with the monomers used in conventional Kevlar production. This is a crucial point, as these waste-derived monomers can be integrated directly into conventional Kevlar production equipment and processes.

Preference is given to North American average values^62^ for chemicals and solvent costs, followed by the latest year bulk purchasing prices. For example, the TCL used for virgin Kevlar production by Dupont US facilities is procured from a Chinese company, and thus, its price is collected directly from the Chinese supplier Web site.^63^

For PET flakes, the national average historical base price is used to calculate the PET flakes price ($0.48/kg), which also includes the mechanical recycling costs (i.e., waste collection and pretreatment). The national average bale price is calculated by taking an average of historical price data from 2014 to 2020,^1^ with the conversion cost as $0.42/kg (Table S5). For years after 2020, the polynomial trend is applied to estimate PET flake costs in 2024. An electricity price of 8.78 cents/kWh, based on the US industrial average for January 2023,^64^ is used. The upcycling process location in this study is assumed to be Virginia, which is the Kevlar manufacturing plant of DuPont. The price details are summarized in Table S5.

### 2.9 Sensitivity Analysis

Sensitivity analysis is useful to examine how assumptions influence the environmental impact and cost. A sensitivity analysis on solvents and byproduct recovery rate, and chemical cost is conducted to evaluate their influence on the economic and environmental impact of Kevlar synthesis. Additionally, reaction parameters, including the yield and temperature for each process, are included in the sensitivity analysis. These parameters are varied to address uncertainties in key process assumptions, as summarized in Table 3.

**Table 3:** Sensitivity Analysis Variables

**Variables**	**Range**
By product recovery rate (%)	varied by ±20% of the initial assumption [54–82][Bibr ref2]
Solvent recovery rate from distillation (%)	varied by ±4.9% of the initial assumption [90.1–99.9][Bibr ref65]
Chemical and solvent cost	varied by ±20% of the initial assumption [0.8–1.2][Bibr ref1]
Reaction yield	varied by ±5% of the initial assumption
Reaction temperature	varied by ±20 °C of the initial assumption

## 3 Results and Discussion

### 3.1 LCA Results of Upcycling PET Waste to Kevlar

Figure 2 shows the cradle-to-gate LCA results for 1 kg of Kevlar production using two methods: (i) chemical upcycling from PET waste and (ii) virgin Kevlar production from petrochemicals. The net impact is calculated by assigning credits of byproducts from the upcycling process. The cutoff approach is applied, where the system boundary begins at waste collection and treatment, and all environmental impacts associated with producing the PET waste are assigned to the original product system. Under this approach, PET waste enters the upcycling system without carrying any upstream environmental burdens. Since virgin PET cannot replace petrochemical feedstocks such as 4-nitroaniline and terephthalic acid in virgin Kevlar production, avoiding virgin PET production is not credited within the system boundary.

**Figure 2:**
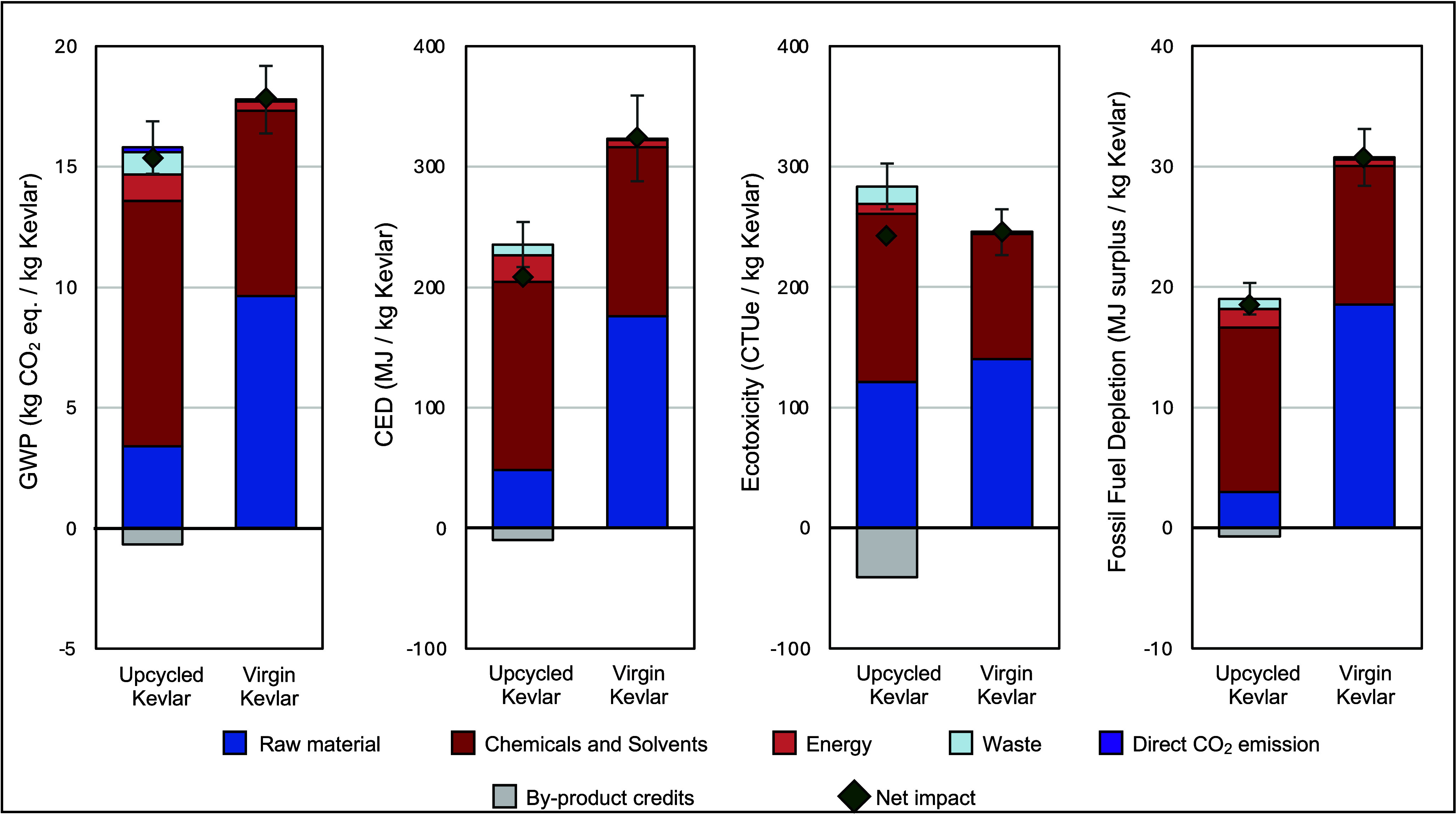
LCA results of 1 kg of upcycled Kevlar and virgin Kevlar production: GWP, CED, ecotoxicity, and fossil fuel depletion.

As shown in Figure 2, the net environmental impacts of the upcycling process are lower than those of virgin Kevlar production across all impact categories. Chemicals and solvents contribute the most to the net environmental impacts: 64% to GWP, 66% to CED, 49% to ecotoxicity, and 71% to fossil fuel depletion. The environmental impact breakdown reveals minimal differences between the upcycling and commercial polymer production processes. Transportation and waste have minor impacts compared to raw materials and chemicals.

The net GWP for chemically upcycling Kevlar from waste PET is estimated to be 15.22 kg of CO_2_ eq/kg of Kevlar, which is approximately 15% lower than conventional fossil-based Kevlar production. This is primarily attributed to less impact from the raw materials, as the PET waste is burden-free, while nitroaniline and terephthalic acid generate emissions. The byproducts, including potassium sulfate and hydrochloric acid, contribute a credit of 0.66 kg of CO_2_ eq/kg Kevlar. The hydrolysis of PET breaks ester bonds, producing one molecule each of potassium terephthalate per repeating unit. Acidification with sulfuric acid to recover terephthalic acid generates potassium sulfate as a byproduct. The chemical upcycling process is credited for recovering materials, offsetting the need to produce an equivalent amount of virgin material.^66^

The LCA result reveals that the upcycled Kevlar has lower impacts compared to conventional polymer synthesis, with a 30% reduction in CED, 1% in ecotoxicity, and 40% in fossil fuel depletion. The higher environmental impact of the virgin polymer originates from the Kevlar polymer synthesis process, which involves consuming a larger volume of NMP compared to the upcycling process. The higher ecotoxicity of conventional polymer production is attributed to 4-nitroaniline, a raw material in PPD preparation known to be toxic to aquatic organisms.^67^ Detailed LCA results for upcycling and commercial production are listed in Table S6.

The Ecoinvent database for terephthalic acid reports a GWP of 1.98 kg CO_2_ eq/kg, which aligns with this study’s findings of 2.22 and 1.76 CO_2_ eq/kg when using KOH and NaOH, respectively. However, there remains a research gap in the environmental impact assessment of PPD, TCL, and PPTA synthesis processes, limiting the comparison of results. The Ecoinvent database indicates a GWP value of 9.26 kg CO_2_ eq/kg PPD synthesized from 4-nitroaniline without solvent inputs, yet the study finds a higher value of 19.1 kg CO_2_ eq/kg PPD, primarily due to chloroform use. This discrepancy underscores the need to optimize chloroform consumption to reduce the GWP associated with PPD and Kevlar polymer production, thereby minimizing environmental impacts across the synthesis process chain.

### 3.2 Alternative Approaches for Open-Loop Recycling

The environmental performance of upcycled Kevlar is assessed under three distinct allocation approaches for open-loop recycling: cut-off, 50/50, and waste valuation. As illustrated in Figure 3, the choice of allocation methodology has an influence, ranging from −2.8 to 16.7% compared to the cutoff method, across different impact categories.

**Figure 3:**
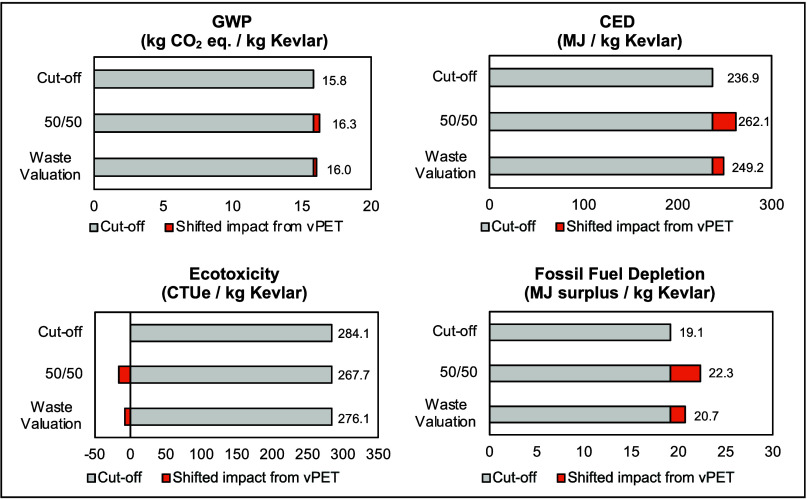
Shifted environmental impacts (GWP, CED, ecotoxicity, and fossil fuel depletion) from the virgin PET life cycle across different allocation methods (cutoff method, 50/50, and waste valuation) for 1 kg of upcycled Kevlar.

For GWP and ecotoxicity, the shifted environmental impact from the virgin PET life cycle is relatively marginal. Specifically, the 50/50 approach results in a 2.7% increase in GWP compared to the cutoff method, while the waste valuation approach shows a 1.3% increase. This marginal impact is due to the comparatively lower GWP associated with virgin PET production compared to that of the chemical upcycling process itself. Ecotoxicity exhibits reductions of 5.8% for the 50/50 approach and 2.8% for waste valuation. These reductions in ecotoxicity are primarily attributed to the higher environmental impact associated with the mechanical recycling of PET waste compared with the production of virgin PET resin, which involves the use of chlorinated chemicals and large amounts of washing water.

Conversely, the CED demonstrates a more substantial shift in impact than the GWP ecotoxicity. The 50/50 approach results in a 10.6% increase, while the waste valuation approach yields a 5.2% increase, compared to the cutoff method. This is predominantly due to the higher electricity consumption associated with the resin production process than the mechanical recycling process, which is allocated to the upcycled Kevlar under these approaches, unlike the cutoff approach, where this burden is not considered. Similarly, fossil fuel depletion shows an increase in impact, with the 50/50 approach resulting in a 16.7% higher impact and the waste valuation approach an 8.2% higher impact than the cutoff method. This increase is primarily driven by the burden associated with p-xylene production, a key raw material for the PET resin, which is allocated to upcycled Kevlar in the 50/50 and waste valuation approaches.

The observed shifts in environmental impacts across different allocation methods highlight the importance of these choices for the recycling of open-loop recycling LCAs. For this study, we chose the cutoff method as our preferred allocation approach. This avoids the complexities of establishing an allocation factor based on fluctuating market conditions for environmental credits, which can be challenging to justify. By assigning no burden to the waste input, the cutoff approach directly focuses on the environmental impacts solely attributable to the upcycling process. This method promotes material recycling for the new production system while avoiding credits that could lead to double-counting or inconsistent interpretations. While alternative methods, such as 50/50 and waste valuation, offer a more detailed perspective by allocating impacts from virgin material production, the cutoff approach provides a clear and consistent framework for evaluating the direct environmental impact of the upcycled product.

### 3.3 Hotspot Analysis

The hotspot analysis identifies the material flow or process with the highest contribution to life cycle impact categories.^68^ In Figure 4, the results reveal that the monomer preparation shows the most GWP and ecotoxicity, particularly due to the roles of solvents, such as chloroform and diethyl ether. The monomer preparation encompasses both PPD and TCL preparation for subsequent PPTA polymer synthesis. Hotspot analysis is especially critical for emerging chemical recycling technologies, which aim to find a sustainable alternative to virgin production and reduce plastic waste. However, the upstream chemical intensity of processes can undermine their environmental benefits. The hotspot analysis can guide process improvement in solvent selection and recovery efficiency to lower the overall environmental impact of the upcycling pathway. The hotspot analysis results for CED and fossil fuel depletion are provided in Figures S2 and S3.

**Figure 4:**
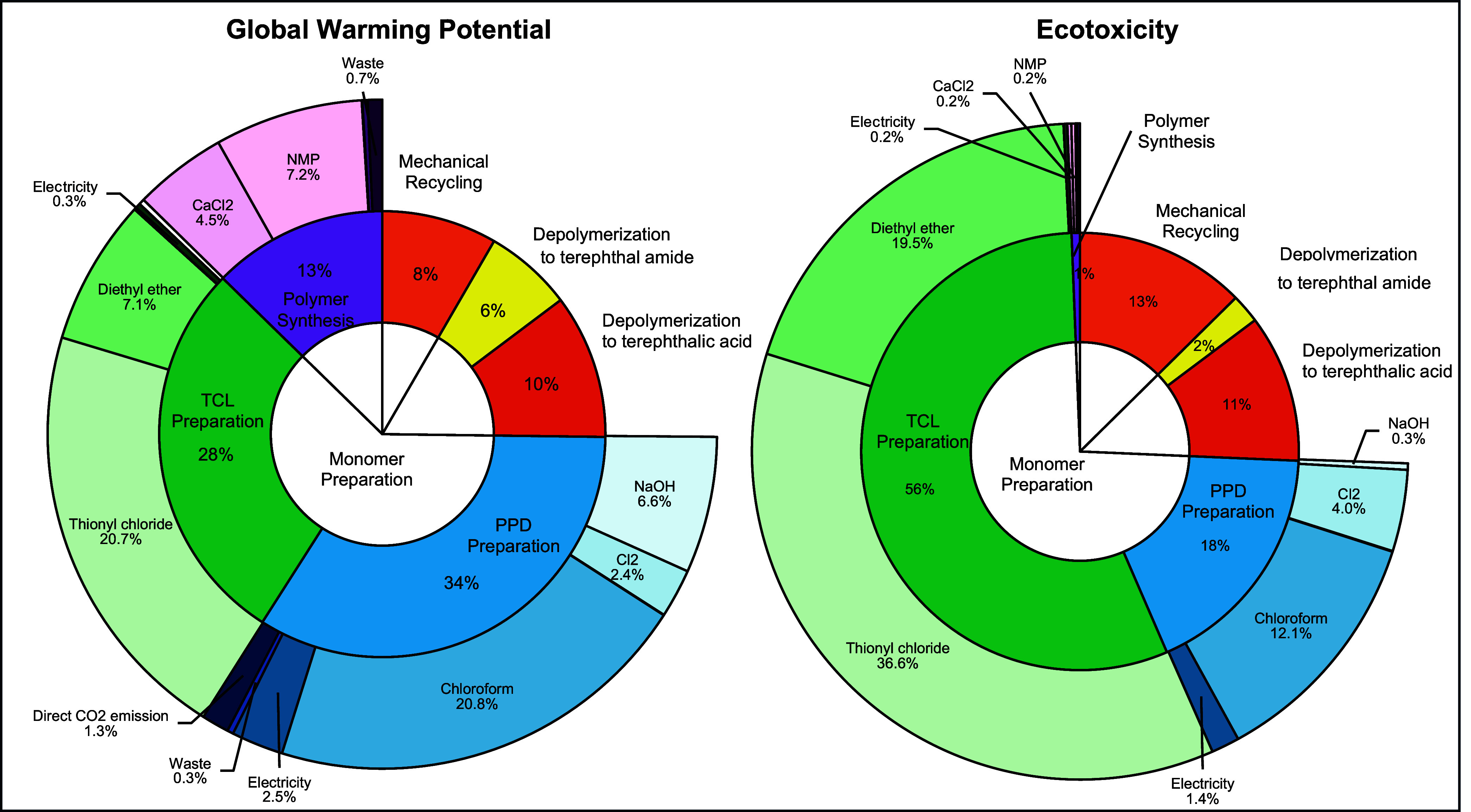
Hotspot analysis results of upcycled Kevlar and virgin Kevlar production: GWP and ecotoxicity.

Regarding GWP, the PPD preparation process is the largest contributor, accounting for 34% of the total impact, followed by the TCL preparation process, which contributes 28%. Chloroform, used in the PPD preparation process for purifying p-phenylenediamine from a terephthalamide mixture, is the largest contributor, accounting for approximately 21% of GWP in the upcycling process. Chloroform’s high GWP is due to the stability and persistence of its carbon-chlorine bonds and the electron-withdrawing effects of chlorine atoms as a volatile organic compound in the atmosphere.^69^ Other contributors to GWP within the PPD preparation process include sodium hydroxide (6.6%), chlorine (Cl_2_) (2.4%), and electricity (2.5%). The results highlight that upstream chemical use, particularly solvent-intensive purification steps, is the major driver of environmental impacts in the upcycling process. Chloroform’s contribution of approximately 21% to the total GWP shows that even a single chemical can significantly raise the carbon footprint. This underscores the need to reduce the use of environmentally intensive solvents through improved recovery, substitution with greener alternatives, or process optimization.

Although the TCL preparation process is the second largest contributor to GWP within the monomer preparation process, it is the most significant process to ecotoxicity (56%), primarily due to the excessive use of thionyl chloride. Thionyl chloride, used in excess during TCL preparation to ensure optimal reaction yields due to its volatility, accounts for 36.6% of the overall ecotoxicity impact. When water or water vapor is exposed, one mole of thionyl chloride produces one mole of sulfur dioxide and two moles of hydrogen chloride, leading to high ecotoxicity.^70,71^

Diethyl ether contributes 7.1% to the total GWP and 19.5% to the ecotoxicity. Diethyl ether is used for purifying the terephthaloyl chloride product after the reaction, with 95% of the solvents being recycled through distillation. Although its impact on GWP is relatively moderate, diethyl ether poses considerable ecotoxicity concerns, largely due to the environmental burden from its upstream production process. Even when solvent recovery rates are high, the use of chemicals with a significant impact on production can still result in considerable environmental impacts. This suggests that prioritizing solvent selection with lower upstream environmental impacts is critical to reducing the overall environmental impact of chemical recycling processes.

Mechanical recycling and depolymerization contribute 8 and 16% to the total GWP, respectively, indicating that chemical recycling introduces higher environmental burdens, despite its potential to regenerate high-performance polymers. While depolymerization itself has a relatively low contribution due to the simplicity of the breakdown reaction, the subsequent monomer and polymer synthesis stages introduce significant upstream impacts driven by solvent use and chemical consumption. These results highlight that the environmental burden of chemical recycling is largely associated with chemical-intensive processes beyond depolymerization. If these upstream impacts are not minimized, then the overall advantages of chemical recycling compared to conventional virgin production may not be achieved. This emphasizes the importance of LCA in capturing the full environmental trade-offs and providing insight into guiding process improvements targeting upstream chemical inputs.

### 3.4 Cost Analysis

Upcycled Kevlar and virgin Kevlar production costs are assessed based on the mass and energy flows derived from the life cycle inventory. As shown in Figure 5, the total cost for upcycled Kevlar is about 23.8% cheaper at 7.03 USD/kg compared with 9.23 USD/kg for virgin Kevlar. The initial mechanical recycling of the plastic waste incurs a minimum cost of 0.60 USD per kg of Kevlar, reflecting its simplicity compared to the complex chemical processes. The cost analysis of PET depolymerization via ammonolysis and hydrolysis shows that the subsequent conversion to terephthalamide and terephthalic acid contributes marginally only to the overall costs, with $0.25/kg and $0.41/kg for Kevlar, respectively. A detailed cost analysis of each intermediate product and Kevlar production is provided in Tables S7 and S8. The estimated cost from depolymerization to the terephthalic acid process is estimated to be $1.18/kg. Previous studies report the minimum selling price of TPA acid from PET to be approximately $1.45/kg, excluding 25% of capital expenditures,^72^ $1.05/kg via methanolysis, and $0.96/kg via glycolysis, while the traditional virgin market price of TPA acid is $1.14/kg. The results suggest that depolymerization is less expensive than fossil-derived acid, even without considering profits and market conditions.

**Figure 5:**
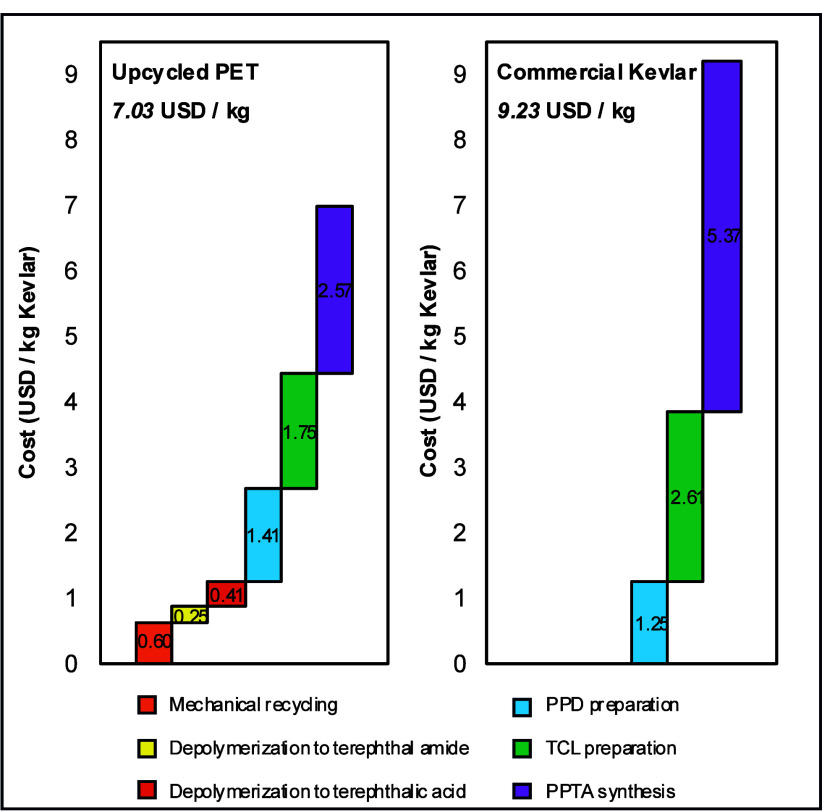
Life cycle cost of upcycled Kevlar and virgin Kevlar.

However, preparing PPD and TCL increases the cost, with additional expenses of $1.41 and $1.75 per Kevlar, respectively. TCL is often preferred for converting carboxylic acids to acid chlorides due to its low cost, making it the most economical method for acid chloride preparation.^73^ Despite this, according to Pfizer’s amidation guide, GSK’s amidation guide, and PubChem, the acid chloride does not meet greenness criteria, being highly corrosive and environmentally toxic.^74,75^ The excessive use of thionyl chloride and diethyl ether primarily drives the high cost of TCL preparation for chemical upcycling. In commercial polymer production, however, the significant cost is mainly due to DuPont’s reliance on TCL imports from a Chinese supplier.^76^

Finally, the synthesis of poly­(p-phenylene terephthalamide) (PPTA) adds $2.57/kg Kevlar, since it only requires mixing in a batch reactor for the PPD and TCL coagulation without solvent extraction.^77^ However, the process for virgin Kevlar is estimated to be $5.37/kg Kevlar. The higher cost in commercial production is attributed to the requirement of twice the amount of NMP, as the reference patent specified 12% to CaCl_2_ by weight, while the upcycling process assumes 16% from the recent patent data. In addition, the elevated cost of NMP, particularly when it is imported from China at $9/kg, contributes to the overall cost.

These findings highlight the trade-off between cost and material quality in recycling methods. Mechanical recycling remains the more economical option but is limited to producing lower-grade products. Chemical recycling, while enabling the conversion to high-value polymers, involves higher costs due to complex chemical reactions and expensive reagents.

### 3.5 Scenario Analysis

Based on the hotspot analysis results, scenarios with modified assumptions on chemicals and solvents are developed to identify how to optimize chemical upcycling, an emerging technology aimed at reducing overall environmental impacts. Figure 6 illustrates the life cycle GWP and cost across various scenarios, including the baseline and modified assumptions for each process. The best combination of these scenarios yields the lowest GWP and cost.

**Figure 6:**
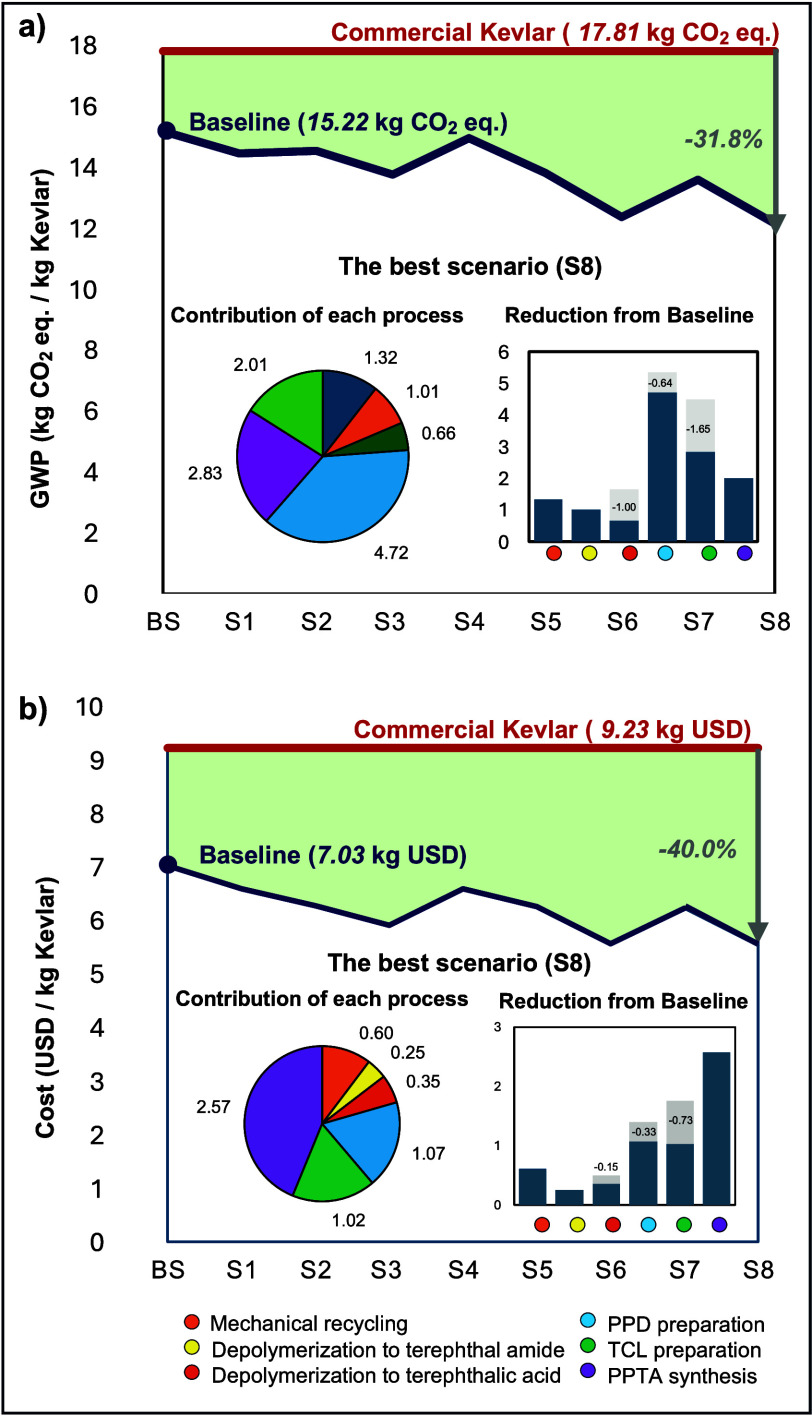
(a) GWP and cost for intermediate products from Kevlar production life cycle stages: PET flake, terephthalamide, terephthalic acid, PPD, TCL, and PPTA. (b) GWP and cost results from scenario analysis compared to virgin Kevlar production and the PET upcycling process. Note: The pie charts illustrate the contribution of each process step, and the bar charts show the reduction from the baseline for the best scenarios. The specific contribution and reduction data are based on the optimized scenarios (S8 for both GWP and cost).

In Scenario 1, the modification involves replacing potassium hydroxide (KOH) with sodium hydroxide (NaOH) to depolymerize PET to terephthalic acid, emphasizing the industrial applicability of the upcycling process. The results indicate that this substitution slightly reduces the GWP by 4.8% and the cost by 6.1% compared with the baseline scenario. A previous study also reports that substituting KOH with NaOH results in similar environmental impacts and positively affects economic outcomes.^78^

Chloroform, used as a purification solvent, contributes 20.8% to the overall GWP due to carbon-chlorine bonds and chlorine’s electron-withdrawal effect, impacting the chemical properties and toxicological profiles of chloromethanes.^69^ In Scenario 2, reducing solvent use lowers the GWP by 4.2% and the cost by 10.6% relative to the baseline, despite the solvent’s low cost limiting potential savings. Compared with commercial production processes, this approach achieves an overall 18.4% reduction in GWP.

Scenarios 3 and 4 adjusted the assumptions of thionyl chloride and diethyl ether based on the hotspot analysis results of the TCL preparation process. These chemicals contribute 28% to total GWP and 56% to ecotoxicity due to excessive consumption, specifically, a 5.3:1 mol ratio of thionyl chloride and 2.5 vol % of diethyl ether, required to achieve the reaction yield, given their high volatility. Scenario 3 assumes a reduced consumption of thionyl chloride to prepare TCL, a 3:1 mol ratio, considering the high capturing yield of thionyl chloride with an industrial-scale reflux system. This adjustment leads to a 9.4% reduction in GWP and a 15.7% reduction in cost savings. In contrast, Scenario 4, which controls diethyl ether consumption, shows less impact, reducing GWP and cost, achieving only 1.5 and 6.2% reduction.

We developed scenarios 5 through 8 by combining the single-factor modifications to achieve greener manufacturing, as shown in Table 2. Scenarios 6 and 7 both involve NaOH for hydrolysis and adjusted chloroform while comparing the impact of thionyl chloride consumption or diethyl ether consumption. The results show that capturing thionyl chloride has a higher reduction potential for both GWP and cost compared to optimizing diethyl ether consumption. The best-case scenario, identified as S8, incorporates all modifications and results in a 31.8% reduction in GWP and a 40.0% reduction in costs compared with the commercial process. Despite these improvements, the monomer preparation and polymer synthesis processes remain the largest contributors to overall emissions, even with the modifications reducing GWP by 0.64 kg CO_2_ eq from PPD preparation and 1.65 kg CO_2_ eq from TCL preparation. The findings highlight the complexity of chemical processes in monomer preparation and polymer synthesis compared to mechanical recycling and depolymerization, underscoring the need for further optimization. The scenarios emphasize the necessity of continued optimization in the upcycling process, particularly as it transitions from an emerging lab-scale technology to a more mature industrial application. Detailed results, including all impact categories and processes, are listed in Table S9.

### 3.6 Sensitivity Analysis

Figure 7 presents the sensitivity analysis for the environmental and cost benefits under the baseline scenario. The solvent recovery rate influences the environment during distillation and the byproduct recovery rate, while the cost is impacted by the prices of solvents and byproducts, as detailed in Table 3.

**Figure 7:**
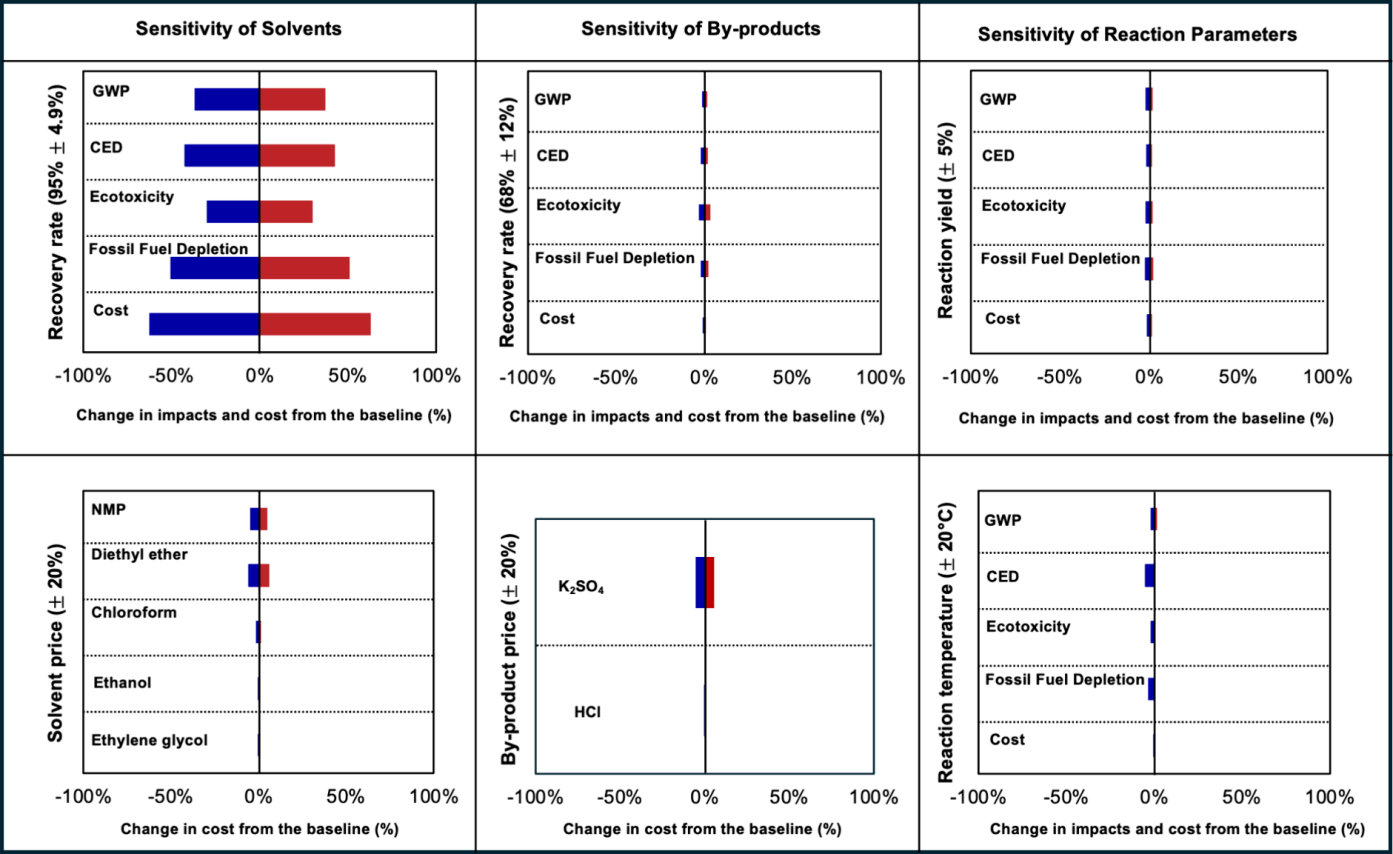
Sensitivity analysis of solvent recovery rate and byproduct recovery rate and reaction parameters (yields and temperatures) on environmental impacts (GWP, CED, ecotoxicity, and fossil fuel depletion) and cost.

Figure 7 shows the effect of solvent and byproduct recovery rates on the GWP, CED, ecotoxicity, and fossil fuel depletion of the upcycling process. In life cycle inventory modeling, a high recovery rate from the distillation process reduces the initial consumption of solvents, affecting all environmental impact categories. Increasing solvent recovery after the distillation process can decrease the GWP by 37% and ecotoxicity by 30%. Sensitivity analysis reveals that more reuse of solvents lowers the environmental impact of chemical processes, particularly from the solvents themselves.^79^ The high sensitivity of the solvent recovery rate and the hotspot analysis findings indicate the need for process modification from a laboratory to an industrial scale to minimize environmental impact while maintaining yield.^55^

The byproduct recovery rate has a marginal impact on all impact categories and costs. The byproducts include ethylene glycol and potassium sulfate from PET depolymerization and acid and hydrochloric acid from PPTA synthesis. The baseline assumes a 68% recovery rate from the waste stream, based on empirical data.^40^ However, a considerably higher recovery rate can be expected, and efficient separation of the byproducts greatly influences both impact and cost.^1,41^ Even though all three chemicals are valuable and can be considered as avoided burdens, their sensitivity is lower than that of solvents, suggesting that the avoided burdens may not always outweigh the additional impacts from energy and chemical consumption.^80^

The analysis reveals that NMP and diethyl ether exhibit higher sensitivity to price changes compared to chloroform, ethanol, and ethylene glycol. NMP’s high sensitivity is due to its high cost ($9/kg), primarily sourced from Chinese suppliers. Diethyl ether’s sensitivity is driven by its substantial bulk usage, accounting for 55% of the total cost in TCL preparation. These findings highlight the important role of solvent pricing in determining the economic viability of the process, particularly for large-scale operations.

The sensitivity analysis for reaction yields and temperature indicates a marginal impact on overall environmental impact categories and cost. This limited sensitivity of environmental impacts to slight changes in reactants and reaction conditions can be attributed to the dominant role of solvents in the process, particularly those consumed in larger quantities during the purification and extraction steps. These high-impact solvents contribute to overall environmental impacts, and their consumption is not directly affected by minor fluctuations in the reaction parameters. Therefore, efforts to reduce the environmental footprint of this emerging technology should prioritize optimizing solvent use, recovery, and selection.

In summary, the sensitivity analysis indicates that byproduct recovery rate and price have marginal impacts on environmental impact and cost compared to solvents. Alternatively, maximizing solvent recovery through distillation and reusing it in the upcycling process is the most effective way to minimize the environmental impact and cost of the upcycling process.

## 4 Conclusions

The present study aims to quantify the environmental impact of chemically upcycling PET to Kevlar and compares it with the environmental impact of virgin Kevlar production in North America. The study develops an inventory of the chemical upcycling process, identifying environmental hotspots for future optimization. Since the upcycling process via ammonolysis and hydrolysis is at the laboratory scale, the life cycle inventory is modeled by scaling up lab data based on patents and published studies. The LCA results show GWP of chemically upcycling Kevlar from waste PET at 15.22 kg CO_2_ eq/kg Kevlar, about 15% lower than conventional methods. Mechanical recycling and depolymerization contribute 8 and 16% of the total GWP, with both showing relatively lower ecotoxicity impacts. Transportation and waste have a minimal impact compared to raw materials and chemicals. Hotspot analysis reveals that monomer preparation is the most critical phase, especially due to solvents like chloroform and diethyl ether. Among the eight evaluated scenarios, Scenario 8 (modifying TPA depolymerization, PPD, and TCL preparation) achieves a 31.8% reduction in GWP and a 40.0% cost reduction compared to the commercial process. Economically, the cost for upcycled Kevlar is estimated at 7.03 USD/kg, compared to 9.23 USD/kg for virgin polymer. Sensitivity analysis indicates that maximizing solvent recovery through distillation and reuse is the most effective way to minimize the environmental impact and cost of the upcycling process. In conclusion, it is determined through this study that the chemical upcycling via ammonolysis and hydrolysis to Kevlar has the potential to mitigate climate change and other environmental impacts compared to virgin Kevlar production while utilizing PET waste. A crucial area for future work is to investigate the feasibility and environmental impacts of replacing high-impact solvents with safer and more sustainable alternatives, as well as to conduct a full Eco-Techno-Economic Analysis (eTEA) of this upcycling pathway. While various methods for techno-economic analysis exist, the recently released ISO/TS 14076 standard provides a structured, ISO-aligned framework for a comprehensive eTEA, combining technical feasibility, economic viability, and environmental impact via LCA. This future work should integrate the environmental impacts with economic indicators such as capital expenditure, profitability, and return on investment to comprehensively evaluate the upcycling technology.

## Supplementary Material



## Notes

Additional life cycle inventory modeling details, hotspot analysis results, scenario analysis results, and cost analysis details (PDF)

## #

Manufacturing Energy Efficiency Research and Analysis Group, Oak Ridge National Laboratory, Oak Ridge, Tennessee 37830, United States
