# Supplementary material for: Life Cycle Assessment of Chemical Upcycling of Postconsumer Polyethylene Terephthalate to Kevlar Polymer
Source: ACS Sustain Chem Eng. 2025 Oct 29;13(44):18924–37. doi: 10.1021/acssuschemeng.5c04073 (PMC12606786; doi:10.1021/acssuschemeng.5c04073)
Supplement: Supplementary file 1 [file sc5c04073_si_001.pdf]

Supporting Information

Life Cycle Assessment of Chemical Upcycling of Post-consumer Polyethylene Terephthalate to Kevlar Polymer

Jaewon Han[1], Preeti Nain[1#], Richard-Joseph L. Peterson[2], Elanna P. Neppel[2], Annick Anctil[1]*

1: Department of Civil and Environmental Engineering, Michigan State University, East Lansing, Michigan 48824, United States.
2: Department of Chemical Engineering, Michigan State University, East Lansing, Michigan 48824, United States.

Present address #: Manufacturing Energy Efficiency Research and Analysis Group, Oak Ridge National Laboratory, Oak Ridge, TN, 37830, United States

**Corresponding author, E-mail: anctilan@msu.edu, Phone: +15174324692.*

Number of pages: 15
Number of Figures: 4
Number of Tables: 9

Table S1. Life cycle inventory of the PET chemical upcycling process

PET to Terephthal amide		Amount	Unit	Assumptions
**Input**	Recycled PET flake	1.149	kg	yield 87%[1]
	Ammonia	0.575	kg	ammonia:PET = 1:2 mass ratio[2]
	Ethylene glycol	2.095	kg	same mole with ammonia based on stoichiometry
	Fresh solvent	0.105	kg	solvent regeneration 95%[3]
**Utility**	Electricity	0.634	kWh	Eq. 2 in section 2.3.2. for 100L reactor[4]
**Product**	Terephtahl amide	1.000	kg	
**Waste**	Chemical waste	0.724	kg	calculated based on mass balance
Terephthal amide to PPD		**Amount**	**Unit**	**Assumptions**
**Input**	Terephthal amide	1.111	kg	yield 90%[2]
	NaOH	2.220	kg	2.22 kg/ PPD[5]
	Cl_2	1.320	kg	1.32 kg/PPD[5]
	DI water	16.281	kg	3M NaOH solution[2]
	Chloroform	48.519	kg	purification solvent 1.49g/cm^3 to water[2]
	Fresh solvent	2.426	kg	solvent regeneration 95%[3]
**Utility**	Electricity	1.996	kWh	Eq. 2 in section 2.3.2. for 100L reactor[4]
**Product**	p-Phenylene diamine	1.000	kg	
**Waste**	Chemical waste	6.402	kg	calculated based on mass balance
	Direct CO_2 emission	0.489	kg	stoichiometry
PET to Terephthalic acid		**Amount**	**Unit**	**Assumptions**
**Input**	Recycled PET flake	1.070	kg	yield 93%[2]
	KOH	0.535	kg	Same mole with 0.5kg NaOH/kg PET[6]
	Ethylene glycol	1.070	kg	1:1 mass ratio of EG to PET[7]
	DI water	0.856	kg	1.25:1 mass ratio EG to water[6]
	H_2SO_4	0.659	kg	0.36ml/g in lab density: 1.83kg/L[2]
**Utility**	Electricity	0.158	kWh	Eq. 2 in section 2.3.2. for 100L reactor[4]
**Product**	Terephthalic acid	1.000	kg	
**By product**	K_2SO_4	0.855	kg	produced same mol with TPA
**Waste**	Chemical waste	2.356	kg	calculated based on mass balance
Terephthalic acid to TCL		**Amount**	**Unit**	**Assumptions**
**Input**	Terephthalic acid	0.818	kg	stoichiometry
	Thionyl chloride	3.125	kg	TPA 3g and thionyl chloride 7ml[8]
	Dimethylformamide	0.002	kg	2.5ml[9]
	Diethyl ether	3.364	kg	2.5vol% to thionyl chloride[9]

**Utility**	Electricity	0.141	kWh	Eq. 2 in section 2.3.2. for 100L reactor[4]
**Product**	Terephthaloyl chloride	1.000	kg	
**Waste**	Chemical waste	3.114	kg	calculated based on mass balance
PPTA synthesis		**Amount**	**Unit**	**Assumptions**
**Input**	Terephthaloyl chloride	0.805	kg	stoichiometry
	p-Phenylene diamine	0.429	kg	stoichiometry
	Calcium chloride	0.838	kg	stoichiometry
	N-Metyl-2-pyrrolidone	5.107	kg	13mol/mol PPD[9]
	DI water	9.917	kg	2:1vol to NMP[9]
**Utility**	Electricity	0.086	kWh	Eq. 2 in section 2.3.2. with 500L reactor [4]
**Product**	**Kevlar**	1.000	kg	
**By product**	HCL	0.145	kg	stoichiometry
**Waste**	Chemical waste	6.40	kg	calculated based on mass balance

Table S2. Life cycle inventory of the commercial Kevlar production

Nitration of MCB		**Amount**	**Unit**	**Assumptions**
**Input**	Monochlorobenzene	0.468	kg	[10]
	Nitric acid	0.262	kg	30% [11]
	Sulfuric acid	0.313	kg	56% [11]
	DI water	0.008	kg	14% [11]
	Ethanol	0.872	kg	7g/100ml ethanol, 95% recycled [3,12]
**Utility**	Electricity	0.041	kWh	[13]
	Heat	4.115	MJ	[13]
**Product**	NCB	1.000	kg	
**Waste**	Chemical waste	0.922	kg	calculated based on mass balance
Amination of 4-NCB		**Amount**	**Unit**	**Assumption**
**Input**	4-Nitrochlorobenzene	1.138	kg	[10]
	Ammonia	1.232	kg	10 mol/mol[11]
	Nitric acid	1.141	kg	2.5mol/mol[14]
	DI water	1.140		water to the nitric acid concentration of 50% [14]
	NaOH	0.306	kg	[10]
**Utility**	Electricity	0.399	kWh	[10]
	Heat	2.094	MJ	[10]
**Product**	4-Nitroaniline	1.000	kg	
**Waste**	Chemical waste	3.96	kg	calculated based on mass balance

Hydrogenation of 4nitroaniline		Amount	Unit	Assumptions
**Input**	4-Nitroaniline	1.276	kg	stoichiometry
	Palladium catalyst	0.000	kg	The 5wt.% Pd for 4-nitroaniline hydrogenation[15]
	Hydrogen gas	0.067	kg	$C_6H_4(NO_2)(NH_2) + 3H_2 \rightarrow C_6H_4(NH_2)_2 + 2H_2O$[15]
	2-Propanol	0.112	kg	50g/100g solvent[16]
	DI water	0.316	kg	0.68mol fraction[15]
**Utility**	Electricity	0.026	kWh	[17]
	Heat	6.434	MJ	[17]
**Product**	p-Phenylenediamine	1.000	kg	100% conversion
**Waste**	Chemical waste	0.773	kg	calculated based on mass balance
TCL preparation		**Amount**	**Unit**	**Assumptions**
**Input**	Terephthalic acid	0.818	kg	stoichiometry
	Thionyl chloride	3.125	kg	TPA:TCL = 0.018mol:0.096mol[8]
	Dimethylformamide	0.002	kg	2.5ml[9]
	Diethyl ether	3.364	kg	2.5vol%[9], 95% recycled[3]
**Utility**	Electricity	0.141	kWh	Eq. 2 in section 2.3.2. for 100L reactor[4]
**Product**	Terephthaloyl chloride	1.000	kg	
**Waste**	Chemical waste	3.114	kg	calculated based on mass balance
PPTA synthesis		**Amount**	**Unit**	**Assumptions**
**Input**	Terephthaloyl chloride	0.653	kg	[18]
	p-Phenylene diamine	0.347	kg	[18]
	Calcium chloride	1.27	kg	[18]
	N-Metyl-2-pyrrolidone	10.36	kg	[3,17]
	NaOH	0.978	kg	[18]
	H_2SO_4	0.013	kg	1wt% to polymer[9]
	DI water	20.12	kg	same mole to the limiting reactant
**Utility**	Electricity	0.16	kWh	Eq. 2 in section 2.3.2. for 500L reactor[4]
**Product**	Kevlar	1.000	kg	
**By product**	HCL	0.117	kg	stoichiometry
**Waste**	Chemical waste	2.673	kg	calculated based on mass balance

Table S3. Reaction parameters for the electricity consumption calculation

**Parameters** [4]		**Unit**
**V**	Reaction mixture volume	m3
C_p	Specific heat capacity of solvent	J/(kg×K)
m_{mix}	Mass of the reaction mixture	kg
ρ_{mix}	Density of the flow	kg/m3
q_{mix}	Volume of the flow	m3
T_r	Reaction temperature	K
T_0	Starting temperature	K
T_{out}	Outside temperature	K
A	Surface area of the reactor	m2
k_a	Thermal conductivity of the insulation material	W/m×K
s	Thickness of the insulation material	m
t	Reaction time	s
η_{heat}	Efficiency of the heater	
	Q heat	MJ
	Q loss	MJ
	Q react	MJ
	Total production	kg
	**Q react/kg product**	**kWh/kg**

Table S4. Calculation on the electricity consumption for the PET chemical upcycling reaction

Parameters	Depolymerization		Precursor preparation		Polymer synthesis
	Ammonolysis	Hydrolysis	PPD	TCL	PPTA
V	0.11	0.11	0.11	0.11	0.55
C_p	3718.20	2427.23	4180.00	583.93	1683.80
m_{mix}	103.76	150.05	126.99	134.14	114.35
ρ_{mix}	1037.61	1500.49	1269.90	1341.36	228.70
q_{mix}	0.10	0.10	0.10	0.10	0.50
T_r	398.15	323.15	353.15	353.15	303.15
T_0	298.15	298.15	298.15	298.15	298.15
T_{out}	288.15	288.15	288.15	288.15	288.15
A	1.27	1.27	1.27	1.27	3.71
k_a	0.04	0.04	0.04	0.04	0.04
s	0.08	0.08	0.08	0.08	0.08
t	7200	72000	900	21600	3600
η_{heat}	0.72	0.72	0.72	0.72	0.72
Q heat	38.58	9.11	29.20	4.31	0.96
Q loss	0.56	1.79	0.04	1.00	0.11
Q react	54.37	15.13	40.61	7.37	1.49
total production	**27.17**	**38.85**	**7.58**	**15.90**	**9.77**
**Q react/kg product**	**0.56**	**0.11**	**1.49**	**0.13**	**0.04**

Figure S1. Mass balance diagram for 1kg of upcycled Kevlar production

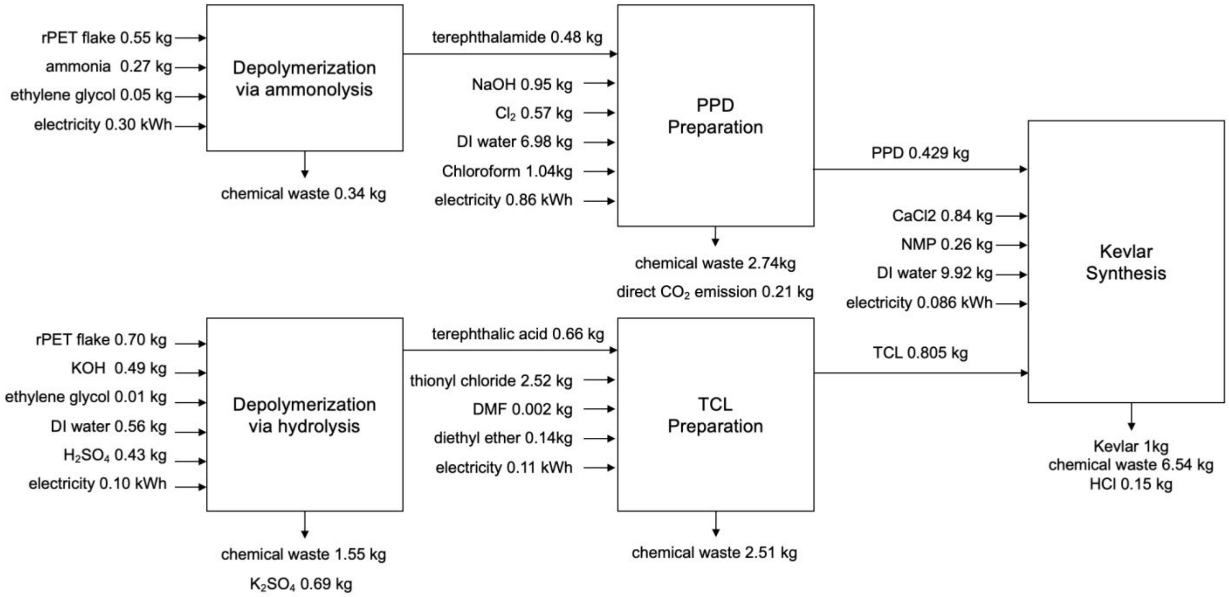

Table S5. PET flake cost estimation based on historical data from 2014 to 2020

Date	National Average Bale Price [7]			Conversion Cost to Flakes [19]	Estimated average PET flake price
	$/kg			$/kg	$/kg
	Low	High	Avg		
5/23/2014	0.46	0.52	0.49	0.42	0.91
04/30/2015	0.28	0.34	0.31	0.42	0.73
08/07/2015	0.29	0.36	0.33	0.42	0.75
02/11/2016	0.17	0.22	0.20	0.42	0.62
07/30/2016	0.23	0.27	0.25	0.42	0.67
11/30/2016	0.2	0.26	0.23	0.42	0.65
08/11/2017	0.33	0.37	0.35	0.42	0.77
01/03/2018	0.28	0.33	0.31	0.42	0.73
07/31/2018	0.35	0.4	0.38	0.42	0.80
01/18/2019	0.31	0.335	0.32	0.42	0.74
06/07/2019	0.32	0.37	0.35	0.42	0.77
01/17/2020	0.18	0.23	0.21	0.42	0.63
04/10/2020	0.18	0.22	0.20	0.42	0.62
09/30/2020	0.12	0.15	0.14	0.42	0.56

The estimated average PET flake price, a critical input for the economic assessment, is detailed in Table S5, drawing upon historical data from 2014 to 2020. This table presents the national average bale price, including its low, high, and average values in $/kg, along with a fixed conversion cost to flakes of $0.42/kg. The estimated average PET flake price is then calculated by summing the average bale price and this conversion cost, providing a transparent basis for the feedstock cost within the upcycling process. For years beyond 2020, a polynomial trend was applied to project these costs.

Table S6. LCIA results breakdown for upcycling and commercial production, including byproducts credits

Category	GWP (kg CO_2 eq./kg Kevlar)		CED (MJ/kg Kevlar)		Ecotoxicity (CTUe/kg Kevlar)		Fossil Fuel Depletion (MJ surplus/kg Kevlar)	
	**Upcycled Kevlar**	**Virgin Kevlar**	**Upcycled Kevlar**	**Virgin Kevlar**	**Upcycled Kevlar**	**Virgin Kevlar**	**Upcycled Kevlar**	**Virgin Kevlar**
Raw material	3.38	9.63	48.49	175.75	121.22	140.03	3.00	18.51
Chemicals and Solvents	10.20	7.70	155.71	140.15	139.64	104.29	13.65	11.50
Energy	1.09	0.37	22.01	6.24	8.29	1.22	1.52	0.56
Transportation	0.09	0.03	1.55	0.34	0.58	0.06	0.12	0.04
Waste	0.91	0.08	9.20	1.31	14.42	0.01	0.86	0.17
Direct emission	0.21							
By-product credits	-0.66		-9.55		-41.11		-0.73	
Total	15.88	17.81	236.96	323.79	284.15	245.61	19.14	30.79
**Net**	15.22	17.81	227.41	323.79	243.04	245.61	8.41	30.79

Figure S2. Hotspot analysis results of CED

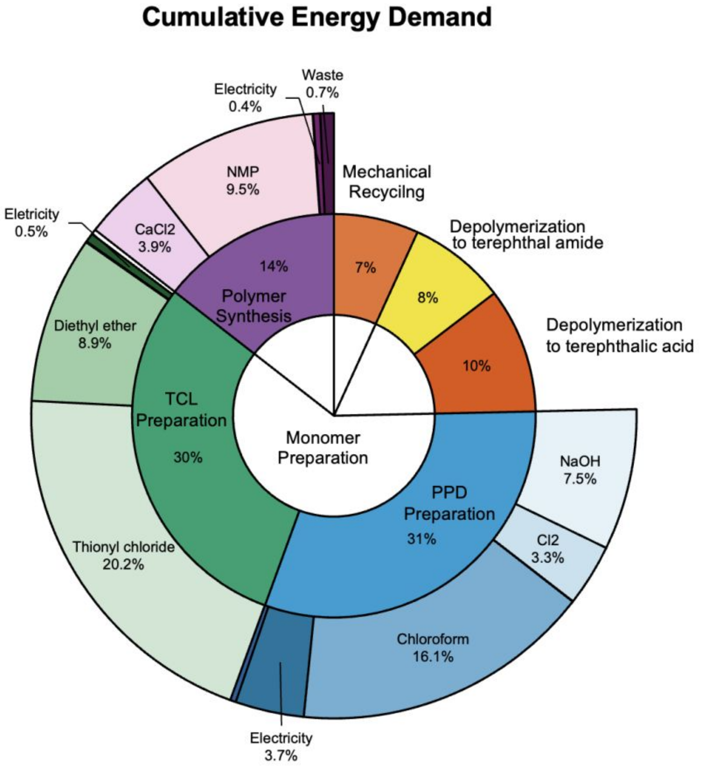

Figure S3. Hotspot analysis results of Fossil Fuel Depletion

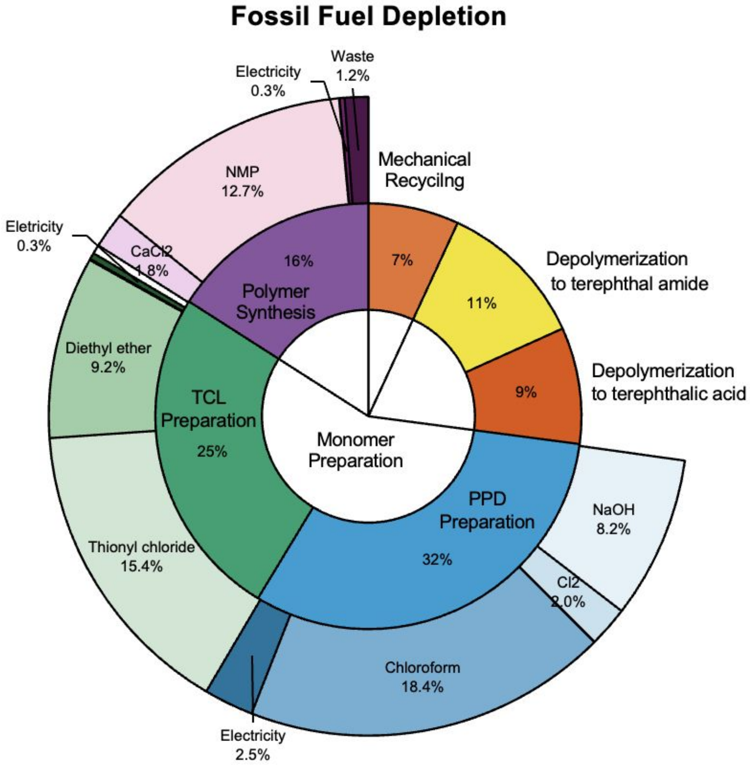

Table S7. Life cycle cost of PET chemical upcycling, including intermediate products

U.S. national average chemical prices in 2024 were collected from Business Analytiq [20], and the industrial average electricity price was obtained from the U.S. Energy Information Administration (EIA) [21].

PET to Terephthal amide		**Price ($/kg)**	**Cost ($/kg product)**
**Input**	Recycled PET flake	0.48	0.555
	Ammonia	0.74	0.425
	Ethylene glycol	0.53	1.110
**Utility**	Electricity	0.087	0.053
**Product**	**Terephthal amide**		**1.089**

PET to Terephthalic acid		**Price ($/kg)**	**Cost ($/kg product)**
**Input**	recycled PET flake	0.48	0.517
	KOH	0.29	0.155
	Ethylene glycol	0.53	0.567
	DI water	0.27	0.000
	H_2SO_4	0.14	0.092
**Utility**	Electricity	0.087	0.013
**Product**	**Terephthalic acid**		**1.184**

Terephthal amide to PPD		**Price ($/kg)**	**Cost ($/kg product)**
**Input**	Terephthal amide	1.089	1.210
	NaOH	0.29	0.644
	Cl_2	0.69	0.911
	DI water	0.27	0.004
	Chloroform	0.64	31.049
**Utility**	Electricity	0.087	0.166
**Product**	**p-phenylene diamine**		**4.487**

Terephthalic acid to TCL		**Price ($/kg)**	**Cost ($/kg product)**
**Input**	Terephthalic acid	1.184	0.969
	Thionyl chloride	0.633	1.978
	Dimethylformamide	1.51	0.004
	Diethyl ether	1.09	0.183
**Utility**	Electricity	0.087	0.012

**Product**	**Terephthaloyl chloride**		**3.145**

PPTA synthesis		Price ($/kg)	Cost ($/kg product)
**Input**	Terephthaloyl chloride	3.145	2.532
	p-Phenylene diamine	4.487	1.923
	Calcium chloride	0.315	0.264
	N-Metyl-2-pyrrolidone	9	2.298
	DI water	0.27	0.003
**Utility**	Electricity	0.087	0.007
**Product**	**PPTA**		**7.03**

Table S8. Life cycle cost of commercial Kevlar production

Commercial PPTA synthesis		Price ($/kg)	Cost ($/kg Kevlar)
**Input**	Terephthaloyl chloride	4	2.61
	p-Phenylene diamine	3.616	1.25
	Calcium chloride	0.315	0.40
	N-Metyl-2-pyrrolidone	9	4.66
	NaOH	0.29	0.28
	H2SO4	0.14	0.00
	DI water	0.27	0.01
**Utility**	Electricity	0.087	0.01
**Product**	**PPTA**		**9.23**

Table S9. Scenario results for GWP, CED, Ecotoxicity, and Fossil fuel depletion

GWP (kg CO_2 eq. / kg Kevlar)									
Process	Base line	S1	S2	S3	S4	S5	S6	S7	S8
Plastic waste treatment	1.06	1.06	1.06	1.06	1.06	1.06	1.06	1.06	1.06
Depolymerization to Amide	2.12	2.12	2.12	2.12	2.12	2.12	2.12	2.12	2.12
Depolymerization to Acid	2.52	1.00	2.52	2.52	2.52	1.00	1.00	1.00	1.00
PPD preparation	12.51	12.51	11.01	12.51	12.51	11.01	11.01	11.01	11.01
TCL preparation	5.57	5.57	5.57	3.79	5.29	5.57	3.79	5.29	3.51
Kevlar synthesis	2.01	2.01	2.01	2.01	2.01	2.01	2.01	2.01	2.01
**Process total**	15.84	14.84	15.20	14.41	15.62	14.20	12.77	13.98	12.55
**Byproduct**	0.66	0.39	0.66	0.66	0.66	0.39	0.39	0.39	0.39
**Net impact**	**15.18**	**14.45**	**14.54**	**13.75**	**14.96**	**13.80**	**12.38**	**13.58**	**12.15**
**CED (MJ / kg Kevlar)**									
Process	Base line	S1	S2	S3	S4	S5	S6	S7	S8
Plastic waste treatment	12.9	12.9	12.9	12.9	12.9	12.9	12.9	12.9	12.9
Depolymerization to Amide	38.9	38.9	38.9	38.9	38.9	38.9	38.9	38.9	38.9
Depolymerization to Acid	36.1	15.7	36.1	36.1	36.1	15.7	15.7	15.7	15.7
PPD preparation	170.8	170.8	87.8	170.8	170.8	87.8	87.8	87.8	87.8
TCL preparation	88.2	88.2	88.2	62.2	83.0	88.2	62.2	83.0	56.9
Kevlar synthesis	34.3	34.3	34.3	34.3	34.3	34.3	34.3	34.3	34.3
**Process total**	237.0	223.5	201.4	216.0	232.7	188.0	167.0	183.7	162.7
**Byproduct**	9.5	6.4	9.5	9.5	9.5	6.4	6.4	6.4	6.4
**Net impact**	**227.4**	**217.1**	**191.9**	**206.4**	**223.2**	**181.5**	**160.6**	**177.3**	**156.3**
**Ecotoxicity (CTUe / kg Kevlar)**									
Process	Base line	S1	S2	S3	S4	S5	S6	S7	S8
Plastic waste treatment	28.6	28.6	28.6	28.6	28.6	28.6	28.6	28.6	28.6
Depolymerization to Amide	12.8	12.8	12.8	12.8	12.8	12.8	12.8	12.8	12.8
Depolymerization to Acid	47.1	1.4	47.1	47.1	47.1	1.4	1.4	1.4	1.4

PPD preparation	118.2	118.2	78.2	118.2	118.2	78.2	78.2	78.2	78.2
TCL preparation	197.2	197.2	197.2	141.1	183.5	197.2	141.1	183.5	127.4
Kevlar synthesis	1.8	1.8	1.8	1.8	1.8	1.8	1.8	1.8	1.8
**Process total**	284.1	254.0	267.0	239.0	273.2	236.9	191.7	225.9	180.7
**Byproduct**	41.1	17.8	41.1	41.1	41.1	17.8	17.8	17.8	17.8
**Net impact**	**243.0**	**236.2**	**225.9**	**197.9**	**232.1**	**219.1**	**173.9**	**208.1**	**162.9**
**Fossil Fuel Depletion (MJ surplus / kg Kevlar)**									
Process	Base line	S1	S2	S3	S4	S5	S6	S7	S8
Plastic waste treatment	1.05	1.05	1.05	1.05	1.05	1.05	1.05	1.05	1.05
Depolymerization to Amide	4.57	4.57	4.57	4.57	4.57	4.57	4.57	4.57	4.57
Depolymerization to Acid	2.57	1.40	2.57	2.57	2.57	1.40	1.40	1.40	1.40
PPD preparation	14.10	14.10	10.00	14.10	14.10	10.00	10.00	10.00	10.00
TCL preparation	6.04	6.04	6.04	4.44	5.60	6.04	4.44	5.60	4.00
Kevlar synthesis	3.05	3.05	3.05	3.05	3.05	3.05	3.05	3.05	3.05
**Process total**	19.14	18.37	17.39	17.85	18.79	16.62	15.33	16.26	14.98
**Byproduct**	0.73	0.47	0.73	0.73	0.73	0.47	0.47	0.47	0.47
**Net impact**	**18.41**	**17.91**	**16.66**	**17.13**	**18.06**	**16.15**	**14.86**	**15.80**	**14.51**

References

(1) Al-Sabagh, A. M.; Yehia, F. Z.; Eshaq, Gh.; Rabie, A. M.; ElMetwally, A. E. Greener Routes for Recycling of Polyethylene Terephthalate. *Egypt. J. Pet.* **2016**, *25* (1), 53–64. https://doi.org/10.1016/j.ejpe.2015.03.001.

(2) Peterson, R.-J. L.; Neppel, E. P.; Peereboom, L.; Trinh, P. A.; Ofoli, R. Y.; Dorgan, J. R. Upcycling Waste PET: I. Ammonolysis Kinetics of Model Dimethyl Terephthalate and the Catalytic Effects of Ethylene Glycol. *ACS Sustain. Chem. Eng.* **2025**, *13* (10), 4120–4131. https://doi.org/10.1021/acssuschemeng.4c10238.

(3) Geisler, G.; Hofstetter, T. B.; Hungerbühler, K. Production of Fine and Speciality Chemicals: Procedure for the Estimation of LCIs. *Int. J. Life Cycle Assess.* **2004**, *9* (2), 101–113. https://doi.org/10.1007/BF02978569.

(4) Piccinno, F.; Hischier, R.; Seeger, S.; Som, C. From Laboratory to Industrial Scale: A Scale-up Framework for Chemical Processes in Life Cycle Assessment Studies. *J. Clean. Prod.* **2016**, *135*, 1085–1097. https://doi.org/10.1016/j.jclepro.2016.06.164.

(5) Zengel, H.-G.; Bergfeld, M. PRODUCTION OF M- AND P-PHENYLENEDIAMINE. 3897498, July 29, 1975. https://patentimages.storage.googleapis.com/63/e0/b3/78998b1c1089f9/US3897498.pdf.

(6) McNeeley, A.; Liu, Y. A. Assessment of PET Depolymerization Processes for Circular Economy. 2. Process Design Options and Process Modeling Evaluation for Methanolysis, Glycolysis, and Hydrolysis. *Ind. Eng. Chem. Res.* **2024**, *63* (8), 3400–3424. https://doi.org/10.1021/acs.iecr.3c04001.

(7) Singh, A.; Rorrer, N. A.; Nicholson, S. R.; Erickson, E.; DesVeaux, J. S.; Avelino, A. F. T.; Lamers, P.; Bhatt, A.; Zhang, Y.; Avery, G.; Tao, L.; Pickford, A. R.; Carpenter, A. C.; McGeehan, J. E.; Beckham, G. T. Techno-Economic, Life-Cycle, and Socioeconomic Impact Analysis of Enzymatic Recycling of Poly(Ethylene Terephthalate). *Joule* **2021**, *5* (9), 2479–2503. https://doi.org/10.1016/j.joule.2021.06.015.

(8) Soni, R. K.; Singh, S. Synthesis and Characterization of Terephthalamides from Poly(Ethylene Terephthalate) Waste. *J. Appl. Polym. Sci.* **2005**, *96* (5), 1515–1528. https://doi.org/10.1002/app.21502.

(9) Neppel, E. P.; Peterson, R.-J. L.; Peereboom, L.; Dorgan, J. R. From Zero to Hero: Polymer Upcycling through Transformation of Waste PET Thermoforms into Kevlar. *ACS Appl. Polym. Mater.* **2025**, *7* (9), 5475–5481. https://doi.org/10.1021/acsapm.5c00191.

(10) Chlorobenzenes Production, Benzene Chlorination, 2024. https://ecoinvent.org/.

(11) Booth, G. Nitro Compounds, Aromatic. In *Ullmann's Encyclopedia of Industrial Chemistry*; Wiley-VCH, Ed.; Wiley, 2000. https://doi.org/10.1002/14356007.a17_411.

(12) Akay, S.; Kayan, B.; Martínez, F. Solubility, Dissolution Thermodynamics and Preferential Solvation of 4-Nitroaniline in (Ethanol + Water) Mixtures. *Phys. Chem. Liq.* **2021**, *59* (6), 956–968. https://doi.org/10.1080/00319104.2021.1888095.

(13) Kim, S.; Overcash, M. Energy in Chemical Manufacturing Processes: Gate-to-gate Information for Life Cycle Assessment. *J. Chem. Technol. Biotechnol.* **2003**, *78* (9), 995–1005. https://doi.org/10.1002/jctb.821.

(14) Veretennikov, E. A.; Lebedev, B. A.; Tselinskii, I. V. Nitration of Chlorobenzene with Nitric Acid in a Continuous Installation. **2001**, *74* (11).

(15) Latypova, A. R.; Lebedev, M. D.; Rumyantsev, E. V.; Filippov, D. V.; Lefedova, O. V.; Bykov, A. V.; Doluda, V. Yu. Amino-Modified Silica as Effective Support of the

Palladium Catalyst for 4-Nitroaniline Hydrogenation. *Catalysts* **2020**, *10* (4), 375. https://doi.org/10.3390/catal10040375.
(16) Jouyban, A. *Handbook of Solubility Data for Pharmaceuticals*; CRC Press: Boca Raton, 2010.
(17) 2-Nitroaniine Production, 2024. https://ecoquery.ecoinvent.org/3.11/cutoff/dataset/2235/documentation.
(18) Vollbracht, L.; Veerman, T. J. PROCESS FOR THE PREPARATION OF POLY P-PHENYLENETEREPHTHALAMIDE. 4308374, December 29, 1981. https://patentimages.storage.googleapis.com/3c/0c/ca/8590f85a5a4e46/US4308374.pdf.
(19) 2 Cleaning Your rPET Stream.
(20) Business Analytiq. Business Analytiq Chemical Price Database. https://businessanalytiq.com/.
(21) Average Price of Electricity to Ultimate Customers by End-Use Sector, by State. https://www.eia.gov/electricity/monthly/epm_table_grapher.php?t=epmt_5_6_a.
